# Targeting Non-classical Myelin Epitopes to Treat Experimental Autoimmune Encephalomyelitis

**DOI:** 10.1038/srep36064

**Published:** 2016-10-31

**Authors:** Xiaohua Wang, Jintao Zhang, David J. Baylink, Chih-Huang Li, Douglas M. Watts, Yi Xu, Xuezhong Qin, Michael H. Walter, Xiaolei Tang

**Affiliations:** 1 Department of Medicine, Division of Regenerative Medicine, Loma Linda University, Loma Linda, California, USA; 2 Division of Infectious Disease, Jinan Infectious Disease Hospital, Shandong University, 22029 Jing-Shi Road, Jinan, 250021, P.R. China; 3 Institute of Medical and Pharmaceutical Sciences, Zhengzhou University, Henan, China; 4 Department of Emergency Medicine, Chang-Gung Memorial Hospital, Linkou Medical Center, Taoyuan, Taiwan; 5 Graduate Institute of Clinical Medical Sciences, School of Medicine, Chang-Gung university, Taoyuan, Taiwan; 6 Department of Biological Sciences, University of Texas at El Paso, El Paso, TX, USA; 7 Musculoskeletal Disease Center, Jerry L. Pettis Memorial Veterans Affairs Medical Center, Loma Linda, California, USA

## Abstract

Qa-1 epitopes, the peptides that bind to non-classical major histocompatibility complex Ib Qa-1 molecules and are recognized by Qa-1-restricted CD8^+^ regulatory T (Treg) cells, have been identified in pathogenic autoimmune cells that attack myelin sheath in experimental autoimmune encephalomyelitis (EAE, an animal model for multiple sclerosis [MS]). Additionally, immunization with such epitopes ameliorates the EAE. However, identification of such epitopes requires knowledge of the pathogenic autoimmune cells which are largely unknown in MS patients. Hence, we asked whether the CD8^+^ Treg cells could directly target the myelin sheath to ameliorate EAE. To address this question, we analyzed Qa-1 epitopes in myelin oligodendrocyte glycoprotein (MOG that is a protein in myelin sheath). Here, we report identification of a MOG-specific Qa-1 epitope. Immunization with this epitope suppressed ongoing EAE, which was abrogated by CD8^+^ T cell depletion. Additionally, the epitope immunization activated the epitope-specific CD8^+^ T cells which specifically accumulated in the CNS-draining cervical lymph nodes. Finally, CD8^+^ T cells primed by the epitope immunization transferred EAE suppression. Hence, this study reveals a novel regulatory mechanism mediated by the CD8^+^ Treg cells. We propose that immunization with myelin-specific HLA-E epitopes (human homologues of Qa-1 epitopes) is a promising therapy for MS.

Multiple sclerosis (MS) is a chronic and debilitating disorder in the central nervous system (CNS). This disease is afflicting more than 2.5 million individuals worldwide. In addition, data suggest that MS global prevalence and incidence rate are increasing^1^. It is believed that the disease is caused by attacks on the myelin sheath by one’s own immune system (autoimmune attacks). Hence, current research efforts focus on developing strategies to arrest the autoimmune attacks. As a result, an array of medications has been approved by the FDA. These medications act to block either the functions of inflammatory molecules or the entrance of immune cells into the CNS^2^. Therefore, the medications do not specifically block the autoimmune attacks on the myelin sheath. Because the immune system uses the same mechanisms to attack the myelin sheath as those to combat health hazards (e.g. infections and cancers), current medications compromise the immune defense mechanism and are still complicated by severe side effects, particularly infections and cancers^3^,^4^.

Accordingly, the ultimate goal of MS therapy is to specifically arrest the autoimmune attacks on the myelin sheath, while sparing global immune defense mechanisms^5^. In principle, antigen-specific therapy is the logical pathway to achieve this goal^5^,^6^. In this regard, the major purpose of an antigen-specific therapy is to specifically instruct potentially pathogenic myelin-specific autoimmune cells, which are responsible for the EAE and MS^7^,^8^,^9^,^10^,^11^, to become myelin-specific regulatory T (Treg) cells. Such Treg cells can then specifically arrest the autoimmune attacks on the myelin sheath without compromising the immune defense mechanisms. However, there is currently no FDA-approved antigen-specific therapy for MS.

Among numerous antigen-specific therapies that are being investigated, the strategies that utilize regulatory Qa-1 epitopes to enhance the function of Qa-1-restricted CD8^+^ Treg cells have unique advantages. In this regard, Qa-1 epitopes are the peptides that bind to non-classical major histocompatibility complex (MHC) Ib Qa-1 molecules and are targets of the Qa-1-restricted CD8^+^ T cells. To support the importance of this Qa-1-epitope-CD8 axis in antigen-specific therapy of MS, recent data have convincingly demonstrated that the dominant role of Qa-1 molecules is presentation of regulatory Qa-1epitopes to the Qa-1-restricted CD8^+^ Treg cells^12^,^13^,^14^,^15^. Indeed, immunization with dendritic cells (DCs) pulsed with the Qa-1 epitopes, derived from pathogenic autoimmune cells, has been shown to specifically suppress EAE through down regulation of the pathogenic autoimmune cells^16^,^17^,^18^,^19^. These animal studies suggest that HLA-E epitopes (the human homologues of murine Qa-1 epitopes) derived from pathogenic autoimmune cells are promising therapeutic agents for MS. However, in MS patients, pathogenic autoimmune cells are largely unknown and hard to determine. Therefore, identification of appropriate HLA-E epitopes in the pathogenic autoimmune cells, if possible, is difficult.

Although pathogenic autoimmune cells have been intensively investigated as the targets of Qa-1-mediated antigen-specific therapy, myelin sheath (i.e. the tissue that is attacked by one’s own immune system in MS patients) has been the target of most antigen-specific therapies^5^. Therefore, we hypothesized that regulatory HLA-E epitopes, specifically located in the myelin sheath, were present and that immunization with such myelin-specific HLA-E epitopes activated the epitope-specific HLA-E-restricted CD8^+^ Treg cells to ameliorate MS. To test this hypothesis, we investigated potential Qa-1 epitopes (the murine homologues of human HLA-E epitopes) in myelin oligodendrocyte glycoprotein (MOG) that is one of the myelin proteins in myelin sheath. Additionally, we studied whether immunization with such epitopes could augment the function of the Qa-1-restricted CD8^+^ T cells to ameliorate EAE. The following is a detailed description of our findings from this study.

## Results

### Portion of CD8^+^ T cells in the CD8^+^ T cell lines reactive to the pool of OLPs (overlapping peptides) covering the whole length of mouse MOG is Qa-1^b^ restricted

Current data suggest that Qa-1-restricted CD8^+^ Treg cells can target pathogenic autoimmune cells^20^ and suppress EAE, an animal model of human MS. In this case, the CD8^+^ T cells achieve the targeting by recognizing regulatory Qa-1 epitopes that are expressed in the myelin-specific pathogenic autoimmune cells^16^,^19^,^21^. However, these regulatory Qa-1 epitopes, though easily identified in animal models, are difficult to define in humans because pathogenic autoimmune cells by themselves are hard to determine in MS patients. This obstacle has prevented further clinical translation of the Qa-1-restricted CD8^+^ Treg cells.

**Figure 1:**
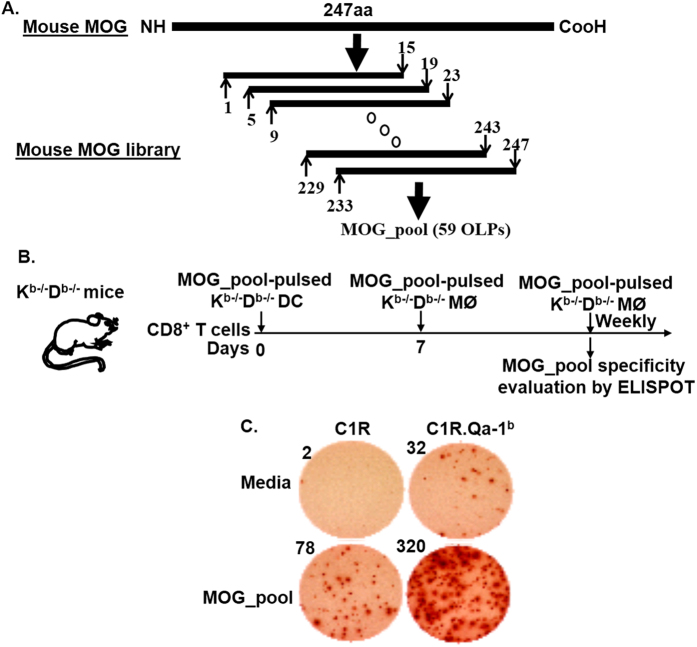
Portion of CD8^+^ T cells reactive to the pool of OLPs covering the whole length of mouse MOG was Qa-1^b^ restricted. (**A**) A schematic view of the mouse MOG OLP library. 15 mer peptides across the whole length of mouse MOG (247aa) and overlapped by 11aa were synthesized. A pool of the 59 OLPs (MOG_pool) at a concentration of 4.2 μg/ml for each peptide was then generated for stimulating CD8^+^ T cells purified from K^b−/−^D^b−/−^ mice. (**B**) Experimental design for “**C**”: CD8^+^ T cells purified from K^b−/−^D^b−/−^ mice were stimulated with the MOG_pool *in vitro* on a weekly basis. Before being stimulated each time, the CD8^+^ T cells were monitored for response to the MOG_pool, using IFN-γ Enzyme-linked ImmunoSpot, in the presence of either C1R or C1R.Qa-1^b^ cells as antigen presenting cells. (**C**) Enzyme-linked ImmunoSpot data on day 7 were shown and were representative of three independent experiments. The number at upper left corner of each well is the absolute number of IFN-γ spot forming cells (SFCs) in the corresponding well (50,000 CD8^+^ T cells/well).

Since pathogenic autoimmune cells in EAE and MS mainly attack myelin sheath, we asked whether Qa-1-restricted CD8^+^ Treg cells could specifically target myelin sheath as well. To answer this question, we proceeded to address if regulatory Qa-1^b^ epitopes which are the targets of Qa-1-restricted CD8^+^ Treg cells were present in mouse MOG since MOG is one of the myelin proteins in myelin sheath. In order to map potential Qa-1^b^ epitopes in mouse MOG, we generated a 15 mer OLP library that covered the whole length of mouse MOG (247 amino acids, or 247aa) from N- to C-termini. All of the OLPs in this library were 15aa in length and overlapped by 11aa. Hence the OLP library contained 59 OLPs in total (Fig. 1, panel A). A pool of the 59 OLPs (MOG_pool), which contained a final concentration of 4.2 μg/ml for each individual peptide, was used to stimulate CD8^+^ T cells purified from K^b−/−^D^b−/−^ mice for generating MOG_pool*-*reactive CD8^+^ T cell lines *in vitro*. Here, we utilized CD8^+^ T cells that were purified from K^b−/−^D^b−/−^ mice because CD8^+^ T cells in these mice were restricted mostly by non-classical MHC Ib molecules including the Qa-1^b^. During *in vitro* stimulations, CD8^+^ T cell lines were monitored weekly for response, using IFN-γ Enzyme-linked ImmunoSpot, to the MOG_pool in the presence of either C1R or C1R.Qa-1^b^ cells. Our data showed that most CD8^+^ T cell lines we generated specifically responded to the MOG_pool in the presence of both C1R and C1R.Qa-1^b^ cells (Fig. 1, panels B,C). However, response to the MOG_pool was much stronger when C1R.Qa-1^b^ cells were present. The data therefore suggested that portion of the CD8^+^ T cells in the lines responded to the MOG_pool in a Qa-1^b^-dependent (or Qa-1-restricted) manner.

### Recognition of multiple OLPs by the MOG_pool-reactive CD8^+^ T cell lines depends on Qa-1^b^

**Figure 2:**
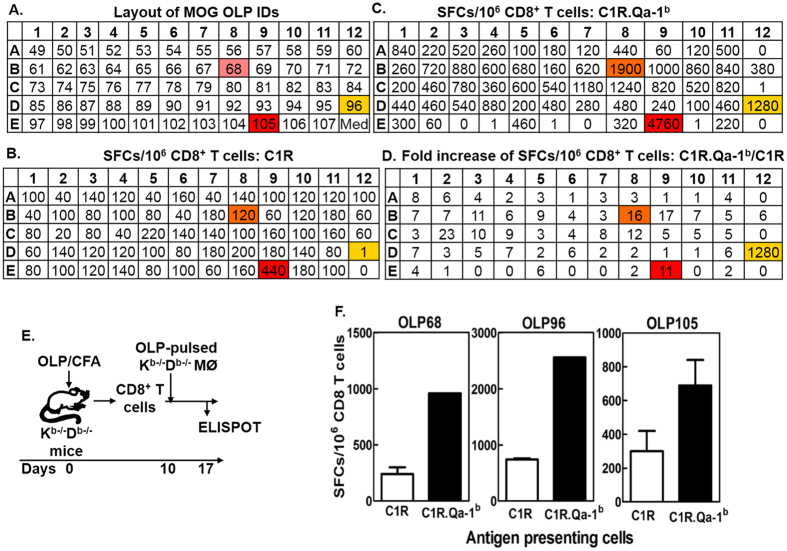
Recognition of multiple OLPs by the MOG_pool-reactive CD8^+^ T cell lines depended on Qa-1^b^. (**A–D**) MOG_pool-reactive CD8^+^ T cell lines were generated as shown in Fig. 1. The 59 individual OLPs were interrogated, using IFN-γ Enzyme-linked ImmunoSpot, for their ability to stimulate the MOG_pool-reactive CD8^+^ T cell lines in the presence of either C1R or C1R.Qa-1^b^ cells. “**A**” Showed the 96-well plate layout of the 59 individual OLPs. Numbers in the wells represented identification codes (IDs) for the 59 individual MOG OLPs, i.e. 49 ~ 107 from N to C termini. “**B**” and “**C**” Showed responses of one representative MOG_pool-reactive CD8^+^ T cell line to the 59 individual OLPs in the presence of either C1R (**B**) or C1R.Qa-1^b^ (**C**) cells. Numbers represented SFCs (spot-forming cells)/10^6^ CD8^+^ T cells. Numbers in “**D**” represented fold increases of SFCs/10^6^ CD8^+^ T cells in the wells that contain C1R.Qa-1^b^ cells (**C**) as compared to C1R cells (**B**), i.e. numbers  =  (SFCs per 10^6^ CD8^+^ T cells in C)/(SFCs per 10^6^ CD8 T cells in B). The highlighted were the top three OLPs that stimulated unequivocal Qa-1^b^-restricted CD8^+^ T cell response. (**E**) Experimental design for “**F**”: K^b−/−^D^b−/−^ mice were immunized with OLP68, OLP96, or OLP105. CD8 T cells purified ten days later were stimulated with the corresponding peptides used for immunization. After one week *in vitro* culture, the CD8 T cells were examined, using IFN-γ Enzyme-linked Immunospot, for specific responses to the corresponding peptides in the presence of either C1R or C1R.Qa-1^b^ cells. (**F**) Representative data from two independent experiments were shown.

We next addressed which individual OLPs in the MOG_pool were recognized by the MOG_pool-reactive CD8^+^ T cell lines in a Qa-1^b^-restricted manner. Thus, the 59 individual OLPs were interrogated individually for their ability to stimulate the MOG_pool-reactive CD8^+^ T cell lines in the presence of either C1R or C1R.Qa-1^b^ cells as antigen-presenting cells (Fig. 2). Data showed that most OLPs provided stronger stimulation of the MOG_pool-reactive CD8^+^ T cell lines in the presence of C1R.Qa-1^b^ cells as compared to C1R cells (Fig. 2, panels B–D). However, only three OLPs, i.e. OLP68, OLP96, and OLP105, consistently stimulated the CD8^+^ T cell lines in a Qa-1^b^-restricted manner. We hence performed a detailed analysis of these three OLPs. Accordingly, K^b−/−^D^b−/−^ mice were immunized individually with the OLP68, OLP96, or OLP105. Ten days later, CD8^+^ T cells were purified from draining lymph nodes and stimulated with the corresponding peptides used for immunization. One week later, the CD8^+^ T cells were examined for response, using IFN-γ Enzyme-linked ImmunoSpot, to the corresponding peptides in the presence of either C1R or C1R.Qa-1^b^ cells. Our data showed that all three OLPs stimulated CD8^+^ T cells in a Qa-1^b^-restricted manner (Fig. 2, panels E,F), suggesting that all three OLPs contained Qa-1^b^ epitopes.

### MOG_196-204_ (hereafter MOG_196_) is the minimal and optimal Qa-1^b^ epitope in OLP105

**Figure 3:**
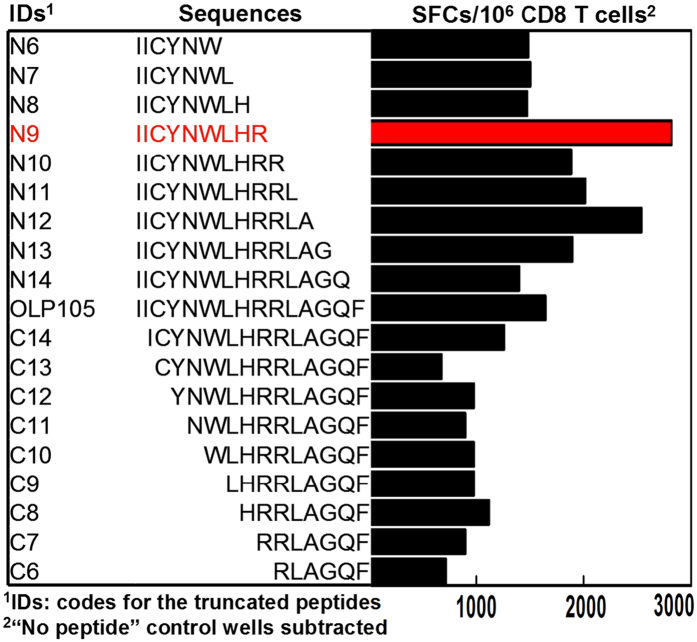
Fine mapping of the minimal and optimal Qa-1^b^ epitope in OLP105. Progressively N- and C-terminally truncated OLP105 peptides were synthesized and interrogated for their ability to stimulate, using IFN-γ Enzyme-linked ImmunoSpot, an OLP105-reactive CD8^+^ T cell line in the presence of C1R. Qa-1^b^ cells. IDs and sequences of the truncated peptides were shown in the left and middle panels respectively. Corresponding bars in the right panel showed IFN-γ spot-forming cells (SFCs) per 10^6^ CD8^+^ T cells in response to corresponding peptides. Highlighted bar and sequence displayed the highest response and stimulating peptide sequence respectively, demonstrating that sequence of the optimal and minimal epitope in OLP105 was IICYNWLHR, i.e. MOG_196-204_ (or MOG_196_). The data was representative of four independent experiments.

Since OLP105 stimulated, in most assays, the highest number of SFCs (spot-forming cells)/10^6^ CD8^+^ T cells in the MOG_pool-reactive CD8^+^ T cell lines in the presence of C1R.Qa-1^b^ cells, we proceeded to further analyze the minimal and optimal Qa-1^b^ epitope in this OLP. Thus, we synthesized progressively N- and C-terminally truncated peptides of OLP105 down to 6 mers because MHC I molecules could present epitopes of 7 ~ 10 mers. These truncated peptides and the original OLP105 were then examined for their ability to stimulate, using IFN-γ Enzyme-linked ImmunoSpot, an OLP105-reactive CD8^+^ T cell line in the presence of C1R.Qa-1^b^ cells. Our data demonstrated that a 9 mer peptide, i.e. IICYNWLHR, was the minimal and optimal epitope in OLP105 (Fig. 3).

### MOG_196_ binds to Qa-1^b^ and activates MOG_196_-specific Qa-1^b^-restricted CD8^+^ T cells *in vitro*

**Figure 4:**
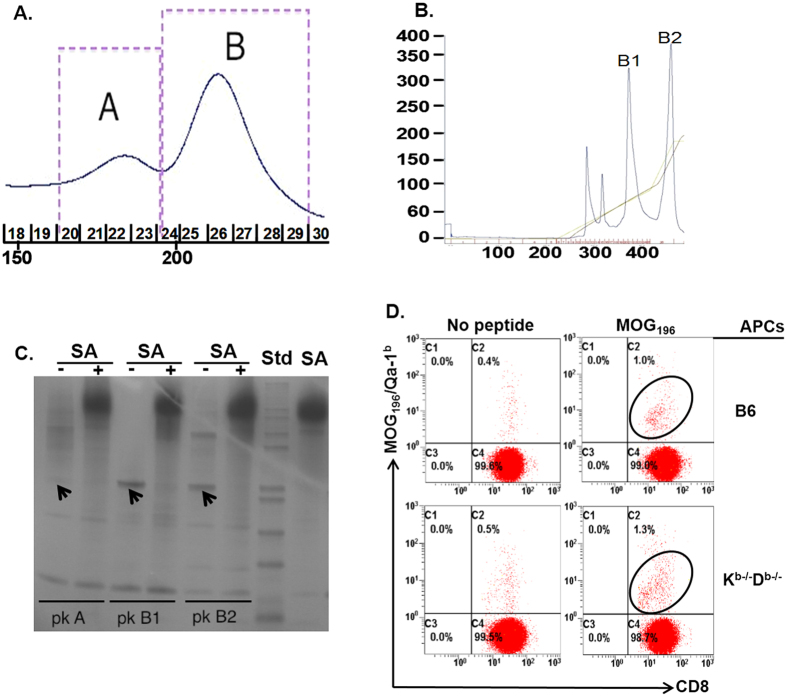
MOG_196_ could bind to Qa-1^b^ and stimulate MOG_196_-specific Qa-1^b^-restricted CD8^+^ T cells. (**A**) MOG_196_ was incubated with recombinant Qa-1^b^ and β2 microglobulin (β2m) in a protein refolding buffer at 10 °C under gentle agitation (60 rpm) for four days. The solution was separated by a size exclusion column. Peak “A” and “B” represented typical non-specific protein aggregates and correctly refolded MOG_196_/Qa-1^b^ monomer respectively. (**B**) Monomers in peak B of panel “A” were further analyzed by an anion exchange chromatography. (**C**) Proteins in peaks “A”, “B1”, and “B2” were biotinylated and portions of the biotinylated proteins were incubated with streptavidin (SA) to examine formation of tetramers. The biotinylated proteins and corresponding tetramers were analyzed in a non-denature protein gel. Arrows show protein bands for MOG_196_/Qa-1^b^ monomers. “−”: without SA; “+”: with SA; “pk A”: peak A; “pk B1”: peak B1; “pk B2”: peak B2; “Std”: a protein standard. (**D**) CD8^+^ T cells were purified from naïve C57BL/6 (B6) mice, individually stimulated weekly with untreated (No peptide) or MOG_196_-pulsed (MOG_196_), either B6 (upper two plots) or K^b−/−^D^b−/−^ (lower two plots) macrophages. The CD8^+^ T cells were analyzed for binding to Qa-1^b^/MOG_196_ tetramer at days 0, 7, and 14. One representative data on day 14 from four individual experiments was shown.

To further confirm that MOG_196_ was a Qa-1^b^ epitope, we next asked whether MOG_196_ could bind to Qa-1^b^ by addressing whether this 9 mer peptide could successfully refold with the recombinant Qa-1^b^ protein *in vitro*. Thus, MOG_196_ was incubated with recombinant Qa-1^b^ protein and β2m at 10 °C for four days. The resulting solution was analyzed in a size exclusion column and displayed a distinct protein peak (Fig. 4, panel A, peak B), suggesting formation of MOG_196_/Qa-1^b^/β2m monomer. When the monomer was further analyzed in an anion exchange column, the monomer displayed two protein peaks (Fig. 4, panel B, peak B1 and B2). To further address potential reasons for the two protein peaks in the anion exchange column, proteins in peaks A, B1, and B2 were biotinylated. Portions of the biotinylated proteins were incubated with Streptavidin that was able to bind four biotinylated proteins to form tetramers. Subsequently, the biotinylated proteins and streptavidin-conjugated tetramers were analyzed in a non-denature protein gel. Data showed that biotinylated proteins in peak A did not show any distinct protein band (Fig. 4, panel C lane 1), supporting that this peak contained mainly non-specific protein aggregates. In contrast, biotinylated proteins in peak B1 displayed a single protein band (Fig. 4, panel C, lane 3), indicating correct formation of MOG_196_/Qa-1^b^/β2m monomer. Interestingly, biotinylated proteins in peak B2 exhibited two protein bands (Fig. 4, panel C, lane 5), suggesting that some proteins in this peak were not correctly refolded. Furthermore, addition of streptavidin successfully conjugated biotinylated proteins in peak B1 and B2 into tetramers (Fig. 4, panel C, lanes 4 and 6). Our data therefore demonstrated that MOG_196_ could bind to Qa-1^b^.

To ask whether MOG_196_ could be presented by antigen-presenting cells to activate MOG_196_-specific Qa-1^b^-restricted CD8^+^ T cells, CD8^+^ T cells purified from C57BL/6 mice were stimulated *in vitro* weekly by MOG_196_-pulsed antigen-presenting cells derived from either K^b−/−^D^b−/−^ or C57BL/6 mice. Data demonstrated that, beginning on day 14 after *in vitro* re-stimulation, MOG_196_/Qa-1^b^ tetramer^+^ cells could be detected in the CD8^+^ T cell lines (Fig. 4, panel D), indicating successful activation of MOG_196_-specific Qa-1^b^-restricted CD8^+^ T cells. In addition, antigen-presenting cells derived from both K^b−/−^D^b−/−^ and C57BL/6 mice were able to present MOG_196_ to activate the MOG_196_-specific Qa-1^b^-restricted CD8^+^ T cells *in vitro* (Fig. 4, panel D).

### Immunization with MOG_196_-pulsed DCs ameliorates MOG_35-55_ EAE

**Figure 5:**
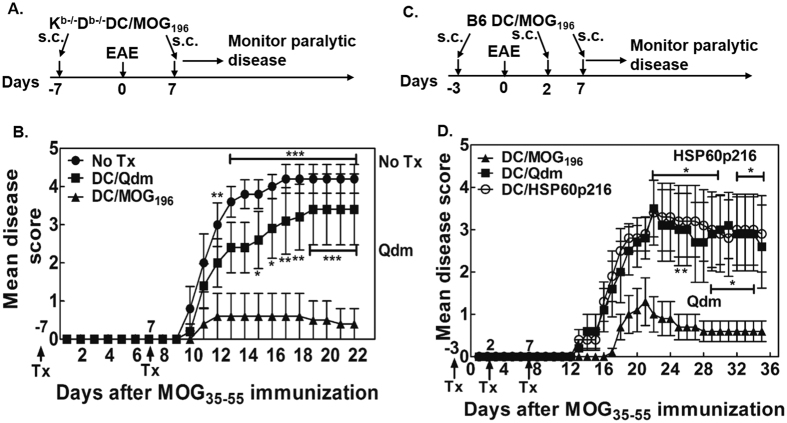
Immunization with MOG_196_-pulsed DCs suppressed MOG_35-55_-induced EAE. (**A**) Experimental design for “B”: C57BL/6 mice were immunized with MOG_35-55_ for EAE. One week before and after the EAE induction, the animals received one of the following subcutaneous treatments: (1) no treatment (No Tx); (2) 1 × 10^6^ Qdm-pulsed K^b−/−^D^b−/−^ DCs (DC/Qdm); (3) 1 × 10^6^ MOG_196_-pulsed K^b−/−^D^b−/−^ DCs (DC/MOG_196_). The mice were then monitored for paralytic disease daily. (**B**) Daily mean disease score was shown. N = 5. *P < 0.05; **P < 0.01; ***P < 0.001. Two-way ANOVA test. (**C**) Experimental design for “D”: C57BL/6 mice were immunized with MOG_35-55_ for EAE on day 0. At days -3, 2, and 7, animals were immunized with 1 × 10^6^ B6 DCs pulsed with Qdm (DC/Qdm), HSP60_p216_ (DC/HSP60_p216_), or MOG_196_ (DC/MOG_196_). The animals were then monitored for paralytic disease daily. (**D**) Daily mean disease score was shown. N = 5. *P < 0.05. Two-way ANOVA test. Concentration of peptides used for pulsing the DCs was 10 μg/ml.

To address whether MOG_196_ is a biologically relevant regulatory Qa-1^b^ epitope, we first asked if immunization with the epitope-pulsed DCs ameliorated MOG_35-55_ EAE. Thus, we immunized C57BL/6 (B6) mice with MOG_196_-pulsed K^b−/−^D^b−/−^ DCs one week before and after the EAE induction. Our data clearly showed that immunization with the MOG_196_- but not the Qdm-pulsed K^b−/−^D^b−/−^ DCs significantly ameliorated the paralytic disease (Fig. 5A,B, Table S1). However, one might have questioned whether immunization with MOG_196_-pulsed wild-type DCs was also able to ameliorate EAE. To evaluate this potential, we immunized C57BL/6 mice with MOG_196_-pulsed B6 DCs on days -3, 2, and 7. On day 0, mice were immunized with MOG_35-55_ for inducing EAE. Our data again showed that immunization with wild-type DCs pulsed with MOG_196_, but not Qdm or HSP60_p216_, significantly ameliorated the paralytic disease (Fig. 5C,D, Table S2).

### Immunization with the MOG_196_-pulsed DCs suppresses ongoing MOG_35-55_ EAE, which is dependent on CD8^+^ T cells

**Figure 6:**
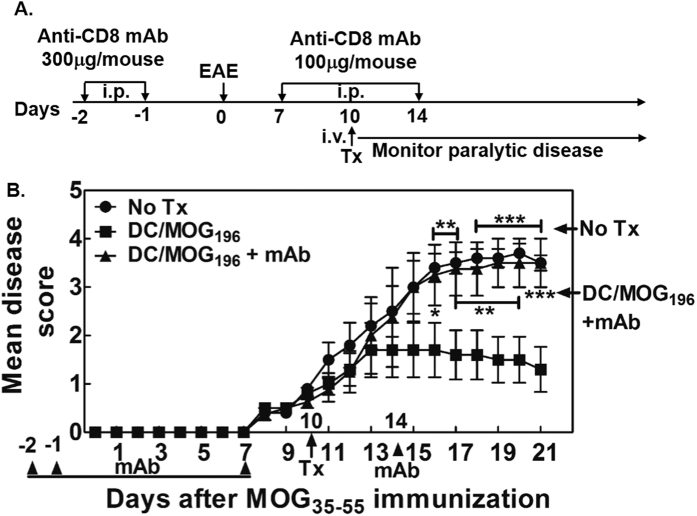
Immunization with MOG_196_-pulsed DCs suppressed ongoing MOG_35-55_-induced EAE, which was dependent on CD8^+^ T cells. (**A**) Experimental design for “B”: C57BL/6 mice were immunized with MOG_55-55_ for EAE on day 0. In one group, the animals were intraperitoneally injected with a monoclonal depleting anti-CD8 antibody (mAb) at days -2, -1, 7, and 14. At day 10, animals received either no treatment (No Tx) or one intravenous injection of mitomycin C-treated C57BL/6 DCs pulsed with MOG_196_ (DC/MOG_196_). Paralytic disease was monitored daily. (**B**) Daily mean disease score was shown. N = 5 (one animal that died from EAE before antibody treatment was excluded from this analysis). *P < 0.05; **P < 0.01; ***P < 0.001. Two-way ANOVA test. Data shown were representative of two independent experiments.

Next, we asked whether immunization with the MOG_196_-pulsed mature B6 DCs could suppress ongoing EAE and whether the disease suppression depended on CD8^+^ T cells. To address the role of the CD8^+^ T cells, we decided to utilize a depleting anti-CD8 monoclonal antibody, i.e. the clone 53–6.7 that had been shown to specifically abrogate the function of CD8^+^ T cells both *in vitro* and *in vivo*^16^,^22^. Hence, C57BL/6 mice were immunized with the MOG_35-55_ for EAE. Ten days later, animals received either no treatment (No Tx) or the MOG_196_-pulsed DCs. Among the animals that received the MOG_196_-pulsed DCs, some animals also received an intra-peritoneal injection of the depleting anti-CD8 antibody at days -2, -1, 7 and 14 to deplete CD8^+^ T cells. Our data showed that, as compared to no treatment, one intravenous injection of 5 × 10^5^ MOG_196_-pulsed B6 DCs robustly suppressed the ongoing paralytic disease (Fig. 6A,B, Table S3). Importantly, depletion of CD8^+^ T cells abrogated the protective effect of the DC/MOG_196_. Therefore, our data demonstrated that suppression of ongoing EAE by the MOG_196_-pulsed DC was dependent on CD8^+^ T cells.

### Immunization with the MOG_196_-pulsed DCs activates CD8^+^ T cells that transfer EAE suppression

**Figure 7:**
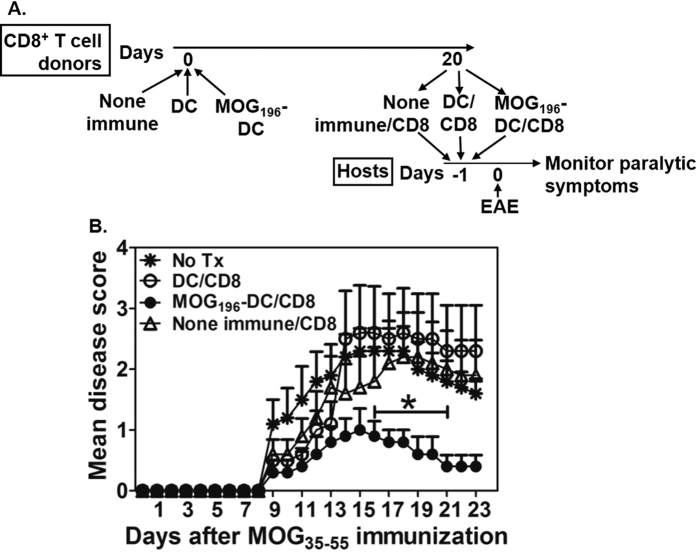
Immunization with the MOG_196_-pulsed DCs activates CD8^+^ T cells that transfer EAE suppression. (**A**) C57BL/6 mice intravenously received no immunization (none-immune), DCs (DC), or MOG_196_-pulsed DCs (MOG_196_-DC). Twenty days later, CD8^+^ T cells from the animals in each group were purified and pooled. Host animals were divided into the following groups: no treatment; DC/CD8 in which each animal received 1 × 10^6^ CD8^+^ T cells from the animals immunized with the DCs; MOG_196_-DC/CD8 in which each animal received 1 × 10^6^ CD8^+^ T cells from the animals immunized with the MOG_196_-pulsed DCs; or none-immune/CD8 in which each animal received 1 × 10^6^ CD8^+^ T cells from the none-immunized animals. On the second day, all animals were immunized with the MOG_35-55_ for inducing EAE and monitored for paralytic symptoms daily. (**B**) Cumulative data of mean disease scores from five animals in each group. *P < 0.05 No Tx vs. MOG_196_-DC/CD8. Mann Whitney test.

To determine whether function of the CD8^+^ Treg cells was augmented by the MOG_196_ immunization, CD8^+^ T cell donor C57BL/6 mice intravenously received no immunization (none-immune), DCs (DC), or MOG_196_-pulsed DCs (MOG_196_-DC). Twenty days later, CD8^+^ T cells were purified from the donor animals and transferred into new host C57BL/6 mice that were immunized with the MOG_35-55_ for inducing EAE on the second day. The animals were monitored for the paralytic symptoms daily (Fig. 7A). Our data showed that transfer of the CD8^+^ T cells from animals immunized with the MOG_196_-pulsed DCs but not the control CD8^+^ T cells significantly suppressed the EAE (Fig. 7B).

### Immunization with MOG_196_-pulsed DCs activates Qa-1^b^/MOG_196_ tetramer^+^ cells that accumulate in the cervical lymph nodes in EAE-bearing animals

**Figure 8:**
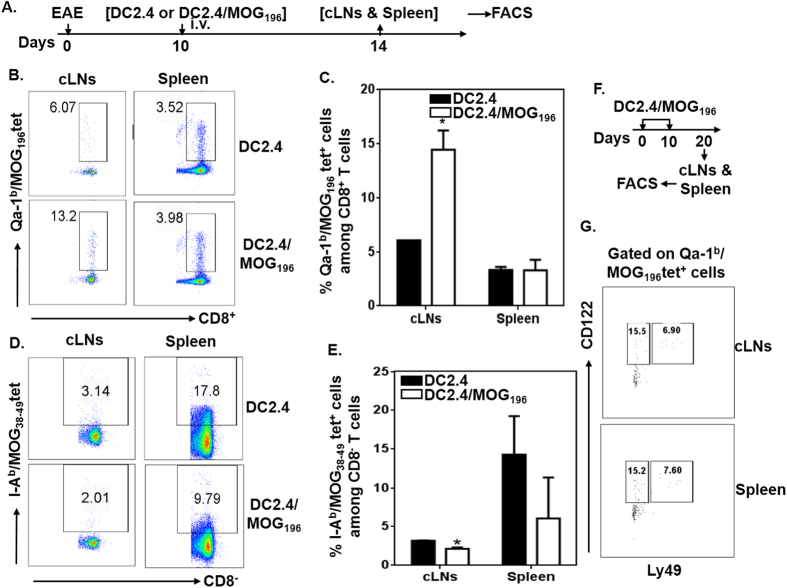
Immunization with DCs pulsed with MOG_196_ activates Qa-1^b^/MOG_196_ tetramer^+^ cells that specifically accumulate in cervical lymph nodes. (**A**) C57BL/6 mice (5 mice/group) were immunized with MOG_35-55_ for EAE. Ten days later, when paralytic symptoms began, animals received one intravenous immunization with mytomycin C-treated DC2.4 or MOG_196_-pulsed DC2.4 (DC2.4/MOG_196_). Four days later, mononuclear cells prepared from spleens and cervical lymph nodes were examined for the presence of Qa-1^b^/MOG_196_ and I-A^b^/MOG_38-49_ tetramer^+^ cells by flow cytometry. (**B**) Representative plots of Qa-1^b^/MOG_196_ tetramer^+^ cells among CD8^+^ T cells in cervical lymph nodes and spleens. (**C**) Cumulative data of Qa-1^b^/MOG_196_ tetramer^+^ cells from five mice. *P < 0.05. Two-way ANOVA test. (**D**) Representative plots of I-A^b^/MOG_38-49_ tetramer^+^ cells among CD8^−^ T cells in cervical lymph nodes and spleens. (**E**) Cumulative data of I-A^b^/MOG_38-49_ tetramer^+^ cells from five mice. *P < 0.05. Two-way ANOVA test. (**F**) Experimental design for “G”: C57BL/6 mice were intravenously immunized with MOG_196_-pulsed DC2.4 cells at days 0 and 10. Ten days after the last immunization, mononuclear cells from cervical lymph nodes and spleens were examined for the expressions of CD122 and Ly49 on Qa-1^b^/MOG_196_ tetramer^+^ CD8^+^ T cells by flow cytometry. (**G**) Representative FACS plots from two independent experiments were shown.

Next, we asked whether immunization with MOG_196_-pulsed DCs indeed activated Qa-1^b^/MOG_196_ tetramer^+^ cells *in vivo* in EAE-bearing animals. Hence, C57BL/6 mice were induced for EAE. Ten days later, when the paralytic disease began, animals received mature DC2.4 cells (DC2.4 is a bone-marrow-derived DC line)^23^ or MOG_196_-pulsed DC2.4 cells (DC2.4/MOG_196_). Four days after the treatments, spleens and cervical lymph nodes were analyzed for the presence of Qa-1^b^/MOG_196_ tetramer^+^ cells. Our data showed that percent of Qa-1^b^/MOG_196_ tetramer^+^ cells in spleens between the two treatments was similar, while percent of tetramer^+^ cells in cervical lymph nodes following the DC2.4/MOG_196_ treatment, as compared to DC2.4 treatment, was significantly elevated (Fig. 8A–C). The data suggested that the Qa-1^b^/MOG_196_ tetramer^+^ cells specifically accumulated in the CNS in EAE-bearing animals. In contrast, I-A^b^/MOG_38-49_ tetramer^+^ cells, which detected MOG_35-55_-reactive pathogenic autoimmune cells, were significantly reduced in cervical lymph nodes (Fig. 8A,D,E). The data were consistent with suppression of ongoing paralytic disease following the treatment with the MOG_196_-pulsed DCs (Fig. 6).

### CD122 and Ly49 are expressed in a portion of the Qa-1^b^/MOG_196_ tetramer^+^ cells

Recent data suggest that Qa-1-restricted CD8^+^ Treg cells are among CD122^+^Ly49^+^ CD8^+^ T cells^12^,^24^,^25^. We hence analyzed expression of these two markers on the Qa-1^b^/MOG_196_ tetramer^+^ cells. Our data showed that about 22.6% of the tetramer^+^ cells expressed CD122 and about 7.2% of the tetramer^+^ cells expressed both CD122 and Ly49 (Fig. 7F,G). Therefore, the data suggest that CD122 and Ly49 are two important markers for Qa-1-restricted CD8^+^ Treg cells. However, additional markers are needed to more precisely identify this subset of CD8^+^ Treg cells.

### MOG_196_ is evolutionarily conversed and unique to MOG

Some known Qa-1-binding peptides, e.g. Qdm and HSP60p216, bind to both Qa-1 and HLA-E^26^. In addition, peptide binding motifs of Qa-1 and HLA-E are similar^26^,^27^. We therefore asked whether MOG_196_ sequence was evolutionarily conserved. Accordingly, blast search of Genbank protein sequence data showed that MOG_196_ sequence was evolutionarily conserved (Fig. S1). Furthermore, sequence of MOG_196_ is specific to MOG and located in the intracellular domain (Fig. S2).

## Discussion

In this article, for the first time, we present evidence for the existence of a regulatory Qa-1^b^ epitope (i.e. MOG_196-204_ or MOG_196_) in myelin oligodendrocyte glycoprotein (MOG) that is a myelin protein in myelin sheath. Immunization with the epitope-pulsed dendritic cells (DCs) suppresses ongoing EAE and activates CD8^+^ T cells that specifically accumulate in cervical lymph nodes. Additionally, CD8^+^ T cells *in vivo* primed by the MOG_196_-pulsed DCs transfer EAE suppression. Hence, this study opens a potential new avenue for the antigen-specific therapy of multiple sclerosis (MS).

The major purpose of antigen-specific therapy is to immunize an individual with pathogenic myelin epitopes (i.e. the myelin epitopes in the myelin sheath targeted by the pathogenic autoimmune cells) under a tolerogenic condition. Such immunization specifically instructs the potentially pathogenic myelin-specific autoimmune cells, which cause the EAE and MS^7^,^8^,^9^,^10^,^11^, to become myelin-specific regulatory T (Treg) cells. These Treg cells can then specifically arrest the autoimmune attacks on the myelin sheath without compromising the immune defense mechanisms. In this regard, one such antigen-specific therapy is the tolerogenic dendritic cell (DC) that is generated *in vitro* under a tolerogenic condition. When pulsed with the pathogenic myelin epitopes, the tolerogenic DCs can induce the epitope-specific Treg cells^28^,^29^,^30^. However, recent data suggest that the tolerogenic DC is unstable in an *in vivo* pro-inflammatory environment and can be converted into a disease-worsening immunogenic DC^31^,^32^,^33^. This instability is hampering clinical translation of the tolerogenic DC^31^,^34^,^35^,^36^,^37^,^38^,^39^. In contrast, we show here that immunogenic DCs can be used to augment the MOG_196_-specific CD8^+^ Treg cells. Therefore, the MOG_196_ therapy does not have the instability concern. An additional advantage is that, unlike the previously reported antigen-specific therapies, the therapeutic strategy described in this report does not depend on the knowledge of the pathogenic myelin epitopes which are still under debate in MS patients.

Although this is the first report on the presence of regulatory Qa-1 epitopes in myelin proteins, previous studies have indicated that immunization with regulatory Qa-1(HLA-E) epitopes is indeed a logical approach for the treatment of EAE. First, animals that were deficient in Qa-1 displayed exaggerated secondary CD4^+^ T cell responses^13^. Second, Qa-1-restricted CD8^+^ T cells in animals whose Qa-1 molecules could not interact with inhibitory NKG2A molecules (Qa-1/R72A mutant mice), as compared with those in wild type animals, showed much stronger suppressive activities^14^. Third, aged animals whose Qa-1 molecules could not interact with CD8 molecules (Qa-1/D227K mutant mice) developed lupus-like autoimmune phenomena^12^. Hence, Qa-1-restricted CD8^+^ T cells are promising sources for antigen-specific therapy.

In addition to the above referred evidence suggesting a dominant regulatory role of Qa-1-restricted CD8^+^ T cells, regulatory Qa-1 epitopes in pathogenic autoimmune CD4^+^ T cells have been described and immunization with these epitopes has been shown to prevent EAE induction. Such epitopes include the p42–50 (GLRLIHYSY) located in the T cell receptor (TCR) Vβ8.2 protein^16^,^17^,^18^ and the LLSWVALFL peptide located in the TCRVβ8.1 protein^21^. Additionally, immunization with the HSP60sp (QMRPVSRAL) located in the leader sequence of the 60 Kda heat shock protein (HSP60) has been shown to prevent the progression of type 1 diabetes in NOD mice^19^. Finally, immunization with the HSP60p216 (GMKFDRGYI) located in the HSP60 has been shown to suppress ongoing collagen-induced arthritis (CIA), suggesting therapeutic potential of the epitope-specific, Qa-1-restricted CD8^+^ Treg cells^25^. All these previously identified Qa-1 epitopes are located in the pathogenic autoimmune cells. It has been proposed that immunization with these epitopes activates Qa-1-restricted CD8^+^ Treg cells that target the pathogenic autoimmune cells to ameliorate autoimmune diseases (e.g. EAE, CIA, and T1D)^19^,^21^,^25^,^40^,^41^. Because the identity of the pathogenic autoimmune cells is hard to determine in patients with MS^42^, clinical translation of these regulatory Qa-1 epitopes is difficult.

As mentioned above, previous studies of Qa-1(HLA-E)-mediated antigen-specific therapy were focused on identification of the regulatory Qa-1 epitopes in the pathogenic autoimmune CD4^+^ T cells. Since efficient entering of CD8^+^ T cells into CNS depends on presentation of CNS-antigens at the blood-brain barrier (BBB)^43^, Qa-1-restricted CD8^+^ Treg cells which target epitopes located in the pathogenic autoimmune CD4^+^ T cells may not efficiently accumulate in the CNS due to a lack of specificity for CNS and therefore may predominantly suppress the pathogenic CD4^+^ T cells in the peripheral lymphoid organs. In contrast, myelin-specific Qa-1-restricted CD8^+^ Treg cells described here will have the advantage to cross the BBB and provide *in situ* suppression of demyelinating inflammation in the CNS. To support this notion, our data showed that, in the course of EAE, MOG_196_-specific, Qa-1-restricted CD8^+^ Treg cells specifically accumulated in the cervical lymph nodes (i.e. the draining lymph nodes for CNS).

With respect to the potential mechanisms underlying the disease suppression mediated by the myelin-specific, Qa-1(HLA-E)-restricted CD8^+^ Treg cells, previous data suggest that the disease suppression involves IFN-γ, perforin, and IL-15^12^,^44^. These data appear to support a direct killing of target cells by the Qa-1-restricted CD8^+^ Treg cells. Since oligodendrocytes normally do not express MHC molecules^45^, it may explain that the MOG_196_-specific Qa-1-restricted CD8^+^ Treg cells do not damage myelin. We propose that the same antigen presenting cell, which phagocytoses myelin, can present both pathogenic and regulatory myelin epitopes to pathogenic CD4^+^ and regulatory CD8^+^ T cells respectively because both epitopes are located in the myelin (Fig. S3). In this regard, recognition of the regulatory Qa-1/HLA-E myelin epitopes by the CD8^+^ Treg cells leads to (*1*) elimination of and/or induction of tolerance in the antigen-presenting cells; (*2*) elimination and/or induction of tolerance in encephalitogenic T cells that have obtained the epitope complexes from antigen-presenting cells via a process called trogocytosis^46^,^47^,^48^. Consequently, activation and proliferation of encephalitogenic T cells are thwarted and autoimmune attacks of myelin sheath are stopped.

A similar recent finding showed that neuroantigen-specific CD8^+^ T cells were regulatory. In this regard, our myelin-specific Qa-1-restricted CD8^+^ Treg cells bear certain similarity to this recently reported neuroantigen-specific CD8^+^ Treg cell subset in terms of their shared specificity to myelin proteins and their dependency on IFN-γ and Perforin^12^,^44^,^49^. However, the neuroantigen-specific CD8^+^ Treg cells differ from myelin-specific Qa-1-restricted CD8^+^ Treg cells in requiring classical MHC I molecules for presentation^50^. In addition, priming of the neuroantigen-specific CD8^+^ Treg cells requires the presence of both the regulatory and pathogenic epitopes in the same myelin protein^50^. This second characteristic suggests that active immunization with such a regulatory neuroantigenic epitope is not a suitable therapy for MS. In contrast, the myelin-specific CD8^+^ Treg cells described here can be primed through active immunization with DCs pulsed with the regulatory epitope alone.

In addition to the neuroantigen-specific CD8^+^ Treg cells, it was shown that glatiramer, an FDA-approved first line medication for MS and a peptide mimicking myelin basic protein^51^, induced HLA-E-restricted CD8^+^ Treg cells in humans^52^. In a MS animal model, glatiramer-mediated suppression of EAE required CD8^+^ T cells and non-classical MIH Ib molecules^53^. Therefore, although glatiramer has multiple functions^54^, induction of Qa-1(HLA-E)-restricted CD8^+^ Treg cells may partially contribute to its therapeutic effect for EAE and MS. Despite glatiramer mimicking myelin basic protein, it has not been demonstrated that it is a strictly myelin-specific antigen. Hence, to our knowledge, the epitope described here is the first myelin-specific regulatory Qa-1 epitope that can actively and independently elicit myelin-specific CD8^+^ Treg cells.

Furthermore, it has been shown that immunization with a myelin epitope via anterior chamber (AC) of the eye (intracameral injection) induces the epitope-specific CD8^+^ Treg cells in the peripheral lymphoid tissues^55^,^56^. Interestingly, suppressive activity of such CD8^+^ Treg cells requires compatibility of the Qa-1 haplotype between the CD8^+^ Treg cells and the target cells^57^, suggesting that Qa-1 molecules are necessary for the CD8^+^ Treg cells to recognize the targets. However, the mechanisms by which the Qa-1 molecules are involved in the induction of such CD8^+^ Treg cells needs further investigation.

Although the myelin-specific Qa-1(HLA-E) epitopes are critical for priming the Qa-1(HLA-E)-restricted CD8^+^ Treg cells which bear the specificity for the myelin sheath, recent data suggest that other strategies may facilitate expansion of these CD8^+^ Treg cells. One potential such agent is the granulocyte macrophage colony stimulating factor (GM-CSF) that has been shown to stimulate the expression of OX40L and Jagged-1 in DCs which promote proliferation of Treg cells^58^,^59^. It would be interesting to know whether the GM-CSF-differentiated DCs, when pulsed with the myelin-specific Qa-1(HLA-E) epitope, can indeed augment expansion of the epitope-specific Qa-1(HLA-E)-restricted CD8^+^ Treg cells.

## Methods

### Mice, cell lines, and reagents

#### Mice

C57BL/6 (B6, female, 8–10 weeks of age, 18–20 g) and *K*^*b*−/−^*D*^*b*−/−^ mice were obtained from Taconic Farms and The Jackson Laboratory (Bar Harbor, Maine, USA) housed in a specific pathogen-free animal facility at the University of Texas at El Paso (UTEP) or Loma Linda University (LLU). All experiments were done in compliance with an Institutional Animal Care and Use Protocol approved by UTEP and/or LLU Animal Care and Use Committee.

#### Cell lines

C1R is a human B lymphoblastoid cell line. C1R.Qa-1^b^ is a stable transfectant that constitutively expresses Qa-1^b^. DC2.4 is a DC line generated from bone-marrow-derived DCs and was kindly provided by Dr. Kenneth L. Rock^23^.

#### Reagents

Peptides used in this study were synthesized at Genemed Synthesis, Inc., San Antonio, TX 78244.

#### IFN-γ Enzyme-linked ImmunoSpot assay

Briefly, plates were coated with an anti-mouse IFN-γ mAb (5 μg/ml in PBS) at 4 °C overnight, blocked with culture medium for two hours at room temperature, and added with desired numbers of CD8^+^ T cells, peptides, and irradiated antigen-presenting cells. After cultured at 37 °C and 5% CO2 for 16 hours, plates were incubated with a biotinylated anti-mouse IFN-γ mAb (2 μg/ml) for two hours followed by streptavidin-conjugated HRP (horseradish peroxidase) for one hour. Finally, plates were incubated with a substrate, monitored for spot development, and washed with distilled water when spots were fully developed. After air-dried, plates were analyzed on an Enzyme-linked ImmunoSpot plate reader for enumerating spots in each well.

#### *In vitro* refolding of a peptide with recombinant Qa-1^b^ protein

Refolding of MOG_196_ with recombinant Qa-1^b^ protein was performed in the NIH tetramer core facility (Emory University, Atlanta, GA)^60^.

#### Induction of MOG_35-55_ -induced EAE

C57BL/6 mice were immunized subcutaneously with either 200 μg MOG_35–55_ emulsified in Incomplete Freund Adjuvant (IFA) supplemented with 250 μg heat-inactivated mycobacterium tuberculosis H37Ra (Difco Laboratories, Michigan, USA). On day 0 and 2, each mouse was administered 150 ng pertussis toxin (Calbiochem, Germany) intraperitoneally. Animals were then assessed for paralytic disease daily using the following scale: “0” = no paralysis, “1” = limp tail, “2” = limp tail and weak gait, “3” = hind limb paralysis, “4” = fore limb paralysis (animals were euthanized at or beyond stage “4”).

#### Generation of bone-marrow-derived DCs

Briefly, bone marrow single cell suspensions (1 × 10^6 ^cells/ml) containing 10 U/ml IL-4 and 100 U/ml GM-CSF were seeded into a 6-well plate (4 ml/well) and cultured at 37 °C, 5% CO2. On day 2, after non-adherent cells were carefully removed, plates were replenished with fresh media and cytokines. On day 4, non-adherent cells containing fresh media and cytokines were transferred into a new 6-well plate. On day 6, cells were replenished with fresh media and cytokines and stimulated with 0.1 μg/ml LPS overnight. On day 7, cells were collected for experiments.

#### Mytomycin C treatment of DCs and peptide pulsing

LPS activated DCs at the concentration of 5 × 10^7^ cells/ml in PBS were treated by mytomycin C (50 μg/ml) for 20 minutes at 37 °C and 5% CO2 (DC2.4 cells were treated for 30 minutes). The treated DCs were then washed three times and adjusted to 5 × 10^6 ^cells/ml in serum-free medium containing 100 μg/ml MOG_196_ or HSP60_P216_ peptide. The cells were pulsed with the peptides for three to four hours at room temperature. After pulsing, the cells were washed once, reconstituted in PBS, and intravenously (or subcutaneously) injected into animals at 200 μl/mouse (5 × 10^5^ cells or 1 × 10^6^ cells/mouse).

#### Multichromatic flow cytometry

Briefly, about 0.5 ~ 1 × 10^6^ cells in a FACS buffer (PBS containing 1% FBS and 0.05% sodium azide) were stained with various fluorescence-conjugated antibodies specific for the desired cell surface proteins or with tetramers at 4 °C for 30 min. The stained cells were washed twice in the FACS buffer before being analyzed on a BD FACSAria II.

#### Statistical analysis

All the statistical analyses were performed using One-Way or Two-Way ANOVA, followed by the SNK-q test or the Dunnett’s Multiple Comparison test. A P-value less than 0.05 was considered statistically significant.

## Additional Information

**How to cite this article**: Wang, X. *et al*. Targeting Non-classical Myelin Epitopes to Treat Experimental Autoimmune Encephalomyelitis. *Sci. Rep.* **6**, 36064; doi: 10.1038/srep36064 (2016).

**Publisher’s note:** Springer Nature remains neutral with regard to jurisdictional claims in published maps and institutional affiliations.

## Supplementary Material

Supplementary Information
