# Supplementary material for: Targeting Non-classical Myelin Epitopes to Treat Experimental Autoimmune Encephalomyelitis
Source: Sci Rep. 2016 Oct 31;6:36064. doi: 10.1038/srep36064 (PMC5086895; doi:10.1038/srep36064)
Supplement: Supplementary Information [file srep36064-s1.pdf]

+SUPPLEMENTAL INFORMATION

Targeting Non-classical Myelin Epitopes to Treat Experimental Autoimmune Encephalomyelitis

Xiaohua Wang[1,4], Jintao Zhang[1,7], David J. Baylink[1], Chih-Huang Li[1,5,6], Douglas M. Watts[2], Yi Xu[1], Xuezhong Qin[1,3], Michael H. Walter[1], Xiaolei Tang[1*]

[1]Department of Medicine, Division of Regenerative Medicine, Loma Linda University, Loma Linda, California, USA; [2]Department of Biological Sciences, University of Texas at El Paso, El Paso, TX, USA; [3]Musculoskeletal Disease Center, Jerry L. Pettis Memorial Veterans Affairs Medical Center, Loma Linda, California, USA. [4]Jinan Infectious Disease Hospital, Shandong University, Shandong, China; [5]Department of Emergency Medicine, Chang-Gung Memorial Hospital, Linkou Medical Center, Taoyuan, Taiwan; [6]Graduate Institute of Clinical Medical Sciences, School of Medicine, Chang-Gung university, Taoyuan, Taiwan. [7]Institute of Medicine, University of Zhengzhou, Henan, China.

*Correspondence should be addressed to Xiaolei Tang, xitang@llu.edu.

Correspondence:

Xiaolei Tang, MD/PhD,
Department of Medicine,
Division of Regenerative Medicine,
Loma Linda University,
Loma Linda, CA. 92354.
Phone: (909) 651-5891;
Fax: (909) 558-0428.
Email: Xitang@llu.edu.

Figure S1.

m LVALIICYNWLHRRLAGQFL

r LVALIICYNWLHRRLAGQFL

C Jacchus LVALIICYNWLHRRLAGQFL

h LVALIICYNWLHRRLAGQFL

Figure S1. MOG_{196} sequence is conserved across species. The data show an alignment of the sequences surrounding MOG_{196} in four different species. “m”: mice; “r”: rats; “C Jacchus”: Callithrix jacchus; “h”: humans.

Figure S2

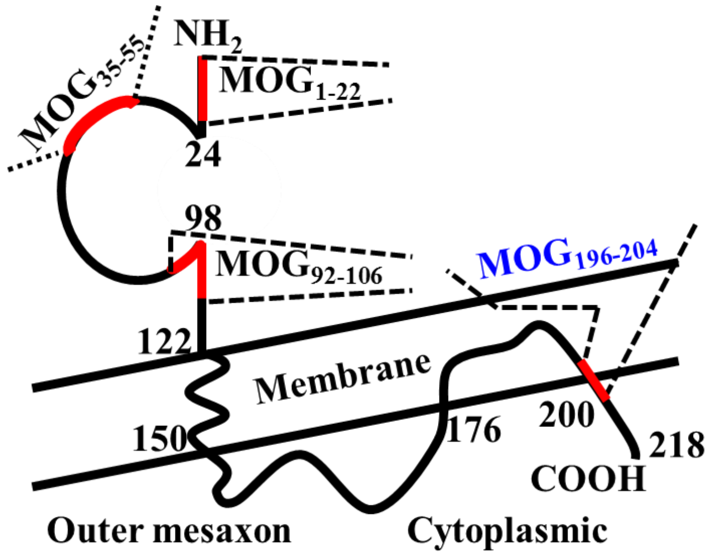

Figure S2. MOG_{196} sequence is located in the intracellular domain of myelin oligodendrocyte glycoprotein (MOG). The three highlighted extracellular epitopes, i.e. MOG_{35-55}, MOG_{1-22}, and MOG_{92-106}, are encephalitogenic (or pathogenic) epitopes. The intracellular MOG_{196} epitope is a regulatory (or protective) $Qa\text{-}1^b$ epitope.

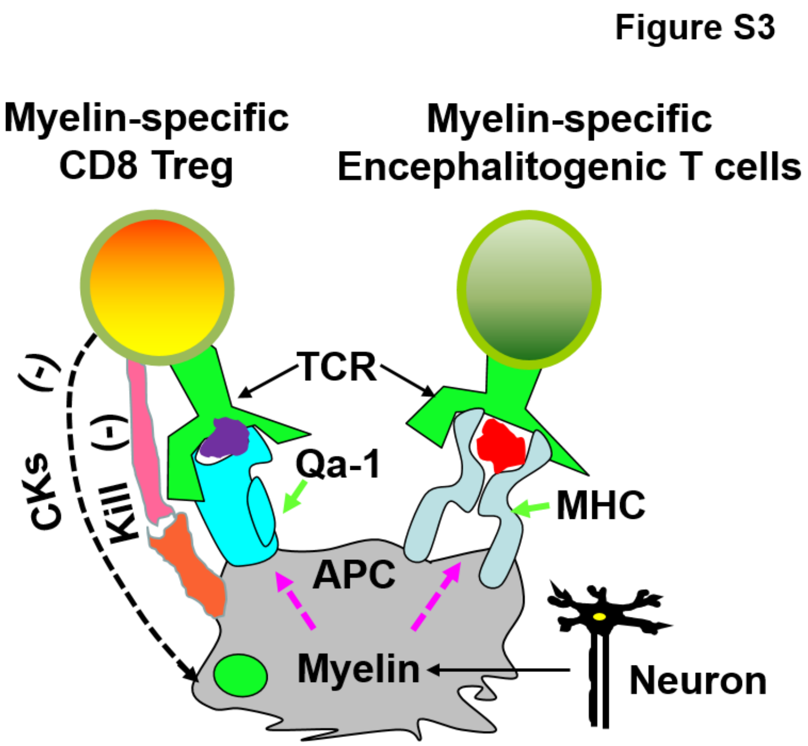

Figure S3. A model of immune regulation mediated by myelin-specific, Qa-1-restricted CD8 Treg. Myelin-specific, Qa-1-restricted CD8 Treg cells can recognize and tolerize/eliminate antigen-presenting cells (APCs) that otherwise activate myelin-specific encephalitogenic T cells in the CNS and/or peripheral lymphoid tissues. Tolerization/elimination of APCs, which present myelin epitopes, is mediated by regulatory cytokines (CKs), inhibitory molecules, or direct cytotoxicity. Consequently, activation of myelin-specific encephalitogenic T cells and autoimmune attacks of myelin sheath are thwarted.

Table S1. Immunization with MOG_{196}-pulsed $K^{b-/-}D^{b-/-}$ DCs suppressed MOG_{35-55}-induced experimental autoimmune encephalomyelitis

Treatments	# of animals with disease/# of total animals (peak scores of individual animals)	Mean days of disease onset	Mean maximal disease score
**No Tx[1]**	**5/5 (4, 5, 4, 5, 4)**	**10.8 ± 0.8**	**4.4 ± 0.5**
**DC/Qdm[2]**	**4/5 (5, 5, 4, 0, 3)**	**11.0 ± 0.8**	**3.4 ± 2.1**
**DC/MOG_{196}[3]**	**1/5 (0, 0, 0, 0, 3)**	**11 ± 0.0**	**0.6 ± 1.3**

[1]No treatment.
[2]Qdm-pulsed $K^{b-/-}D^{b-/-}$ DCs.
[3]MOG_{196}-pulsed $K^{b-/-}D^{b-/-}$ DCs.

Table S2. Immunization with MOG_{196}-pulsed C57BL/6 DCs suppressed MOG_{35-55}-induced experimental autoimmune encephalomyelitis

Treatments	# of animals with disease/# of total animals (peak scores of individual animals)	Mean days of disease onset	Mean maximal disease score
**DCs/$HSP60_{p216}$[1]**	**5/5 (5, 5, 3.5, 2.5, 2)**	**14.2 ± 1.0**	**3.1 ± 0.3**
**DCs/Qdm[2]**	**5/5 (3.5, 3.5, 3.5, 2.5, 2)**	**15 ± 1.1**	**3.2 ± 0.3**
**DCs/MOG_{196}[3]**	**3/5 (2.5, 2.5, 1.5, 0, 0)**	**16.7 ± 0.3**	**1.3 ± 0.6**

[1]$HSP60_{p216}$-pulsed C57BL/6 DCs.
[2]Qdm-pulsed C57BL/6 DCs.
[3]MOG_{196}-pulsed C57BL/6 DCs.

Table S3. Suppression of ongoing MOG_{35-55}-induced experimental autoimmune encephalomyelitis by MOG_{196} immunization was dependent on $CD8^+$ T cells

Treatments	# of animals with disease/# of total animals (peak scores of individual animals)	Mean days of disease onset	Mean maximal disease score
**No Tx[1]**	**5/5 (3.5, 4, 4, 4, 3)**	**8.4 ± 0.9**	**3.7 ± 0.4**
**DCs/ MOG_{196}[2]**	**5/5 (3, 1.5, 3, 0.5, 0.5)**	**8 ± 0**	**1.7 ± 1.3**
**DCs/MOG_{196} + mAb[3]**	**4/5 (3, 5, 3, 3)[4]**	**8.3 ± 0.5**	**3.5 ± 1**

[1]No Treatment.
[2]MOG_{196}-pulsed C57BL/6 DCs.
[3]MOG_{196}-pulsed C57BL/6 DCs + anti-CD8 mAb.
[4]One animal that died before treatment was excluded from this analysis.
